# Descending controls modulate inflammatory joint pain and regulate CXC chemokine and iNOS expression in the dorsal horn

**DOI:** 10.1186/1744-8069-10-39

**Published:** 2014-06-20

**Authors:** Fiona B Carr, Sandrine M Géranton, Stephen P Hunt

**Affiliations:** 1 Department of Cell and Developmental Biology, University College London, London WC1E 6BT, UK

**Keywords:** RVM, Joint pain, 5-HT, Mu opioid receptor, CXCL9, CXCL10, iNOS, CXCR3

## Abstract

**Background:**

Descending control of nociceptive processing, by pathways originating in the rostral ventromedial medulla (RVM) and terminating in the dorsal horn, contributes to behavioural hypersensitivity in a number of pain models. Two facilitatory pathways have been identified and are characterized by serotonin (5-HT) content or expression of the mu opiate receptor. Here we investigated the contribution of these pathways to inflammatory joint pain behaviour and gene expression changes in the dorsal horn.

**Results:**

Selective lesion of the descending serotonergic (5-HT) pathway by prior intrathecal administration of 5,7-dihydroxytryptamine attenuated hypersensitivity at early time points following ankle injection of CFA. In a separate study ablation of the mu opioid receptor expressing (MOR+) cells of the RVM, by microinjection of the toxin dermorphin-saporin, resulted in a more prolonged attenuation of hypersensitivity post CFA. Microarray analysis was carried out to identify changes in dorsal horn gene expression associated with descending facilitation by the MOR+ pathway at 7d post joint inflammation. This analysis led to the identification of a number of genes including the chemokines *Cxcl9* and *Cxcl10*, their common receptor *Cxcr3*, and the proinflammatory gene *Nos2* (inducible nitric oxide synthase, iNOS).

**Conclusions:**

These findings demonstrate that joint pain behaviour is dependent in part on descending facilitation via the RVM, and identify a novel pathway driving CXC chemokine and iNOS expression in the dorsal horn.

## Background

Joint pain is a significant clinical problem [1-3]. In conditions such as osteoarthritis and rheumatoid arthritis the extent of peripheral joint pathology does not always correlate with pain levels [4,5] suggesting chronic joint pain may be due in part to changes within the central nervous system. A number of studies have investigated the role of peripheral sensitisation in joint pain in animal models [6-12]. However central sensitisation of the dorsal horn has also been reported to contribute to mechanical hypersensitivity [8,13-15]. Recently a role for dorsal horn immune cells in joint pain has also been described [16,17].

Descending facilitation via the rostral ventromedial medulla (RVM) has been shown to be an important factor in a number of animal models of chronic pain [18]. Two largely independent descending facilitatory pathways have been described. One originates from 5-HT (serotonin) positive cell bodies within the RVM and has been shown to release 5-HT into the dorsal horn, and the other is characterized by expression of the mu opioid receptor (MOR+) on cell bodies within the RVM [19]. MOR+ cells are believed to correspond to the electrophysiologically defined, pro-nociceptive ON cell population [19,20]. Depletion of MOR+ RVM neurons by administration of the selective neurotoxin dermorphin-saporin has been shown to prevent the maintenance of neuropathic pain behaviour [21-23]. Similarly, manipulation of the descending 5-HT pathway has been shown to attenuate pain in animal models. Thus reducing 5-HT synthesis within the RVM [24], or depletion of 5-HT containing descending fibres within the spinal cord [25,26], have been shown to attenuate hypersensitivity following both neuropathic injury and cutaneous inflammation.

To date, the role of descending facilitation in joint pain behaviour has not been investigated but it is likely that the MOR+ and 5-HT pathways also contribute to behavioural hypersensitivity in models of joint pain. In addition, little is known regarding the molecular mechanisms within the dorsal horn that are modulated by descending facilitation. The aim of the present study was to investigate the contribution of descending facilitation to behavioural hypersensitivity in a model of ankle joint inflammation, using selective lesion of the MOR+ or 5-HT pathways. To better understand the molecular mechanisms in the dorsal horn underlying descending facilitation, microarray analysis was also carried out to identify dorsal horn genes regulated by the MOR+ descending pathway.

## Results

### Lesion of descending 5-HT fibres attenuates mechanical hypersensitivity at 1d and 2d post CFA

**Figure 1:**
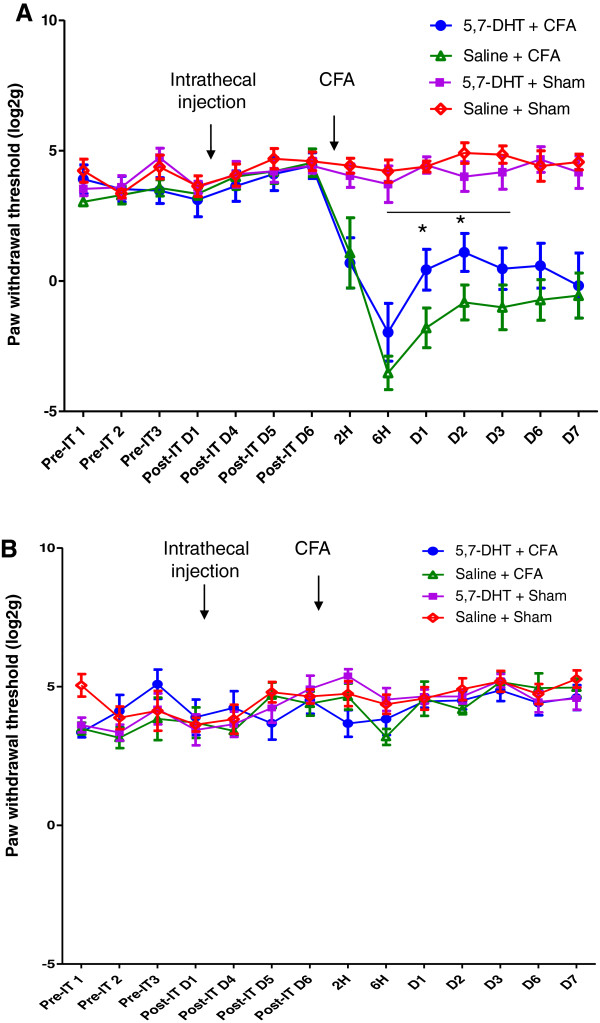
**Attenuation of mechanical hypersensitivity by 5,7-DHT treatment. A).** Attenuation of mechanical hypersensitivity in 5,7-dihydroxytryptamine treated rats following ankle joint inflammation. Ipsilateral paw withdrawal thresholds were significantly higher in 5,7-DHT treated animals compared with saline controls, at 1d and 2d following CFA injection. Pre-IT indicates the baseline prior to intrathecal injection, post-IT indicates baseline after IT injection. **B).** Paw withdrawal thresholds of the contralateral hindpaw were not altered by CFA injection or by 5,7-DHT pre-treatment. Data is presented as log2 (paw withdrawal threshold in g) and mean ± SEM. Line indicates significant 5,7-DHT x CFA interaction in ANOVA, * indicates p < 0.05, LSD post hoc tests. n = 6–7 per group.

**Figure 2:**
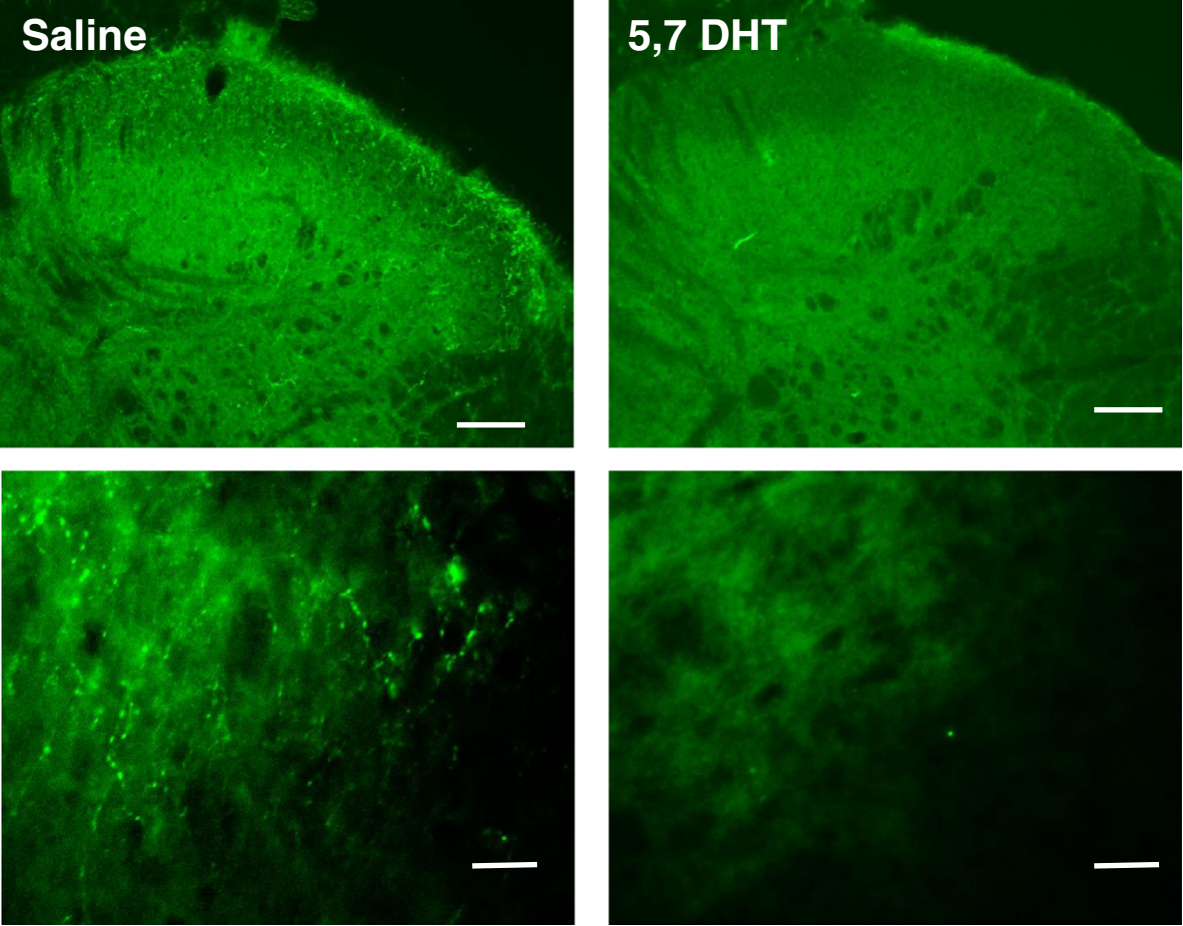
**Immunohistochemistry confirms depletion of 5-HT fibres by 5,7-DHT treatment.** Representative images indicate that 5,7-DHT treatment leads to a complete loss of 5-HT punctate immunoreactivity within the dorsal horn. Top row x 5 magnification, scale bar indicates 400 μm, bottom row x 10 magnification, scale bar indicates 200 μm. Tissue was taken at 7d post CFA injection (13d following 5,7-DHT depletion).

To determine the contribution of the descending 5-HT to mechanical hypersensitivity following ankle joint inflammation, animals were divided into two groups, receiving intrathecal injection of 5,7-DHT or saline. These were subdivided into groups which underwent CFA injection or sham procedure. Paw withdrawal thresholds were measured prior to 5,7-DHT or saline treatment, and monitored up to 7d post CFA injection. Three-way analysis of variance (ANOVA) with repeated measures was carried out, with time as the within-subject factor and intrathecal injection (5,7-DHT or saline) and CFA injection (CFA or sham) as the between-subjects factors (n = 6–7). All animals that received CFA injection developed mechanical hypersensitivity of the ipsilateral hindpaw from 2 h post injection (p < 0.001, F (1, 22) = 87.3). Examining the time window from 6 h to 3d indicated a significant interaction of intrathecal injection x CFA (p = 0.031, F (1, 22) = 5.3), and subsequent LSD post hoc tests indicated a significant effect of 5,7-DHT treatment on CFA behaviour at 1d and 2d after injection (p = 0.01 and p = 0.037) (Figure 1A).Paw withdrawal thresholds of the contralateral hindpaw were also measured. No main effect of CFA injection was identified, indicating contralateral mechanical threshold is unaffected by CFA injection (Figure 1B).To confirm ablation of 5-HT fibres from the dorsal horn, 5-HT immunohistochemistry was carried out on a subset of animals at the end of behavioural experiments. The lumbar L4-L6 region of the spinal cord from a total of 8 rats (2 from each treatment group, in each of the two experimental batches) were sectioned and stained for 5-HT. In all 5,7-DHT treated animals it was found that as expected, 5-HT positive punctae were almost completely absent within the dorsal horn, compared with saline injected controls in which extensive 5-HT labelling was observed. Representative images of 5,7-DHT treated and saline control animals are shown in Figure 2.

### Lesion of descending MOR+ cell pathway attenuates mechanical hypersensitivity from 1-7d post CFA

**Figure 3:**
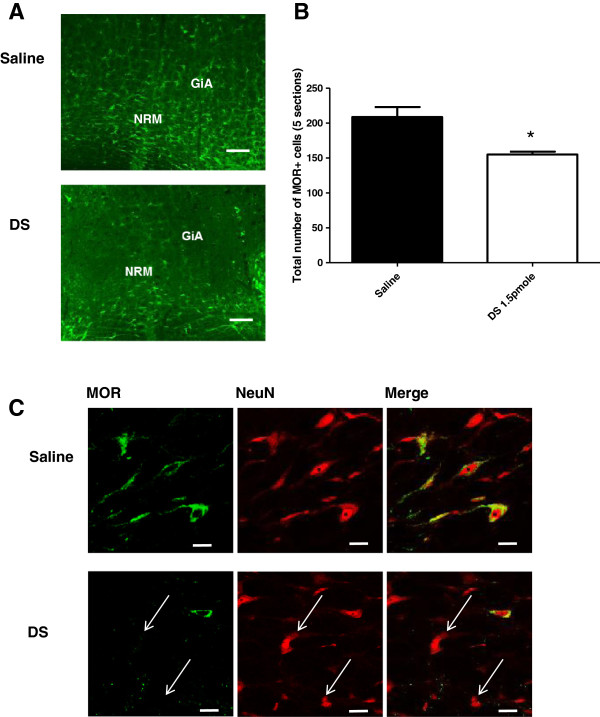
**Immunohistochemistry confirms depletion of MOR+ neurons by dermorphin-saporin microinjection to the RVM. A).** Representative images illustrate MOR+ expression in dermorphin-saporin and saline control animals (approximately −10.5 mm from Bregma). Depletion of MOR+ cells can be observed in the bilaterally injected dermorphin-saporin (DS, 3pmole/96 ng) animal. Scale bar indicates 200 μm. Nucleus raphe magnus (NRM) and nucleus reticularis gigantocellularis (GiA), components of the RVM are shown. **B).** Cell counts indicate a significant decrease in MOR+ cell number in animals treated with 1.5pmole dermorphin-saporin (DS). Cell counts were calculated as the sum of the top five sections per animal, and data shown here as mean ± SEM per group. n = 3 – 4, *p = 0.022, independent samples t-test. **C).** Representative single plane confocal images of MOR immunohistochemistry in the 1.5pmole dermorphin-saporin group and saline control. Double labelling with NeuN indicated that although many MOR+ neurons are depleted at this dose, some surviving MOR- neurons remain in the region, indicated by white arrows. Scale bars indicate 25 μm.

**Figure 4:**
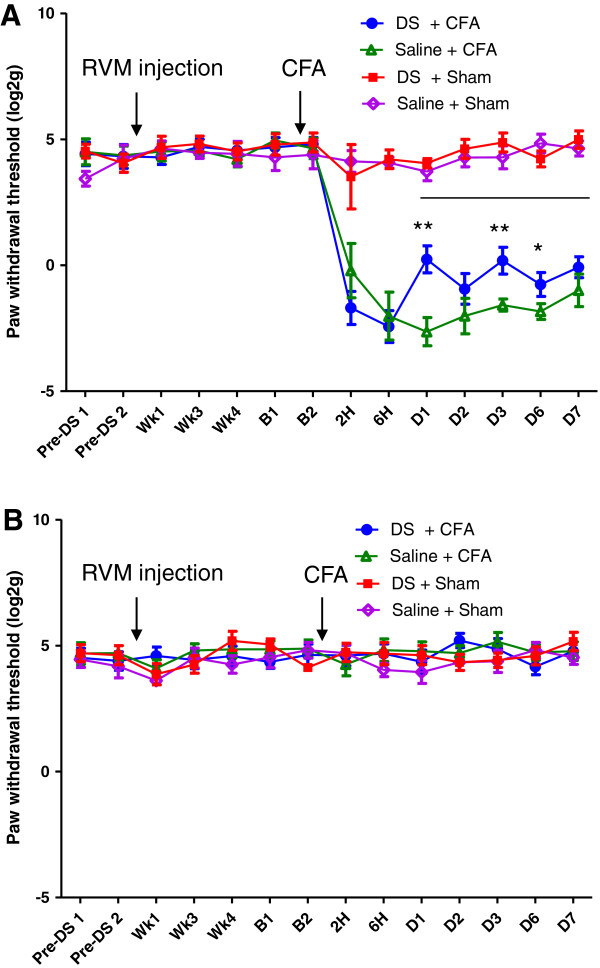
**Attenuation of mechanical hypersensitivity by dermorphin-saporin microinjection. A).** Attenuation of mechanical hypersensitivity of the ipsilateral hindpaw in dermorphin-saporin (DS, 1.5pmole in total, 0.75pmole each side) pretreated rats following ankle joint inflammation. Paw withdrawal thresholds were significantly higher in the dermorphin-saporin group than in saline injected controls from 1 to 7d post CFA injection. Line indicates overall effect of DS treatment from 1d to 7d post CFA in ANOVA with repeated measures. *p < 0.05 and **p < 0.01 with LSD post hoc tests. There was no effect of pre-treatment on baseline paw withdrawal thresholds. **B).** Paw withdrawal thresholds of the contralateral hindpaw were not altered by CFA injection or by dermorphin-saporin pre-treatment, p > 0.05, three-way ANOVA with repeated measures. Data is presented as log2 (paw withdrawal threshold in g) and mean ± SEM, n = 8 in CFA groups, n = 6 /7 in sham groups.

**Figure 5:**
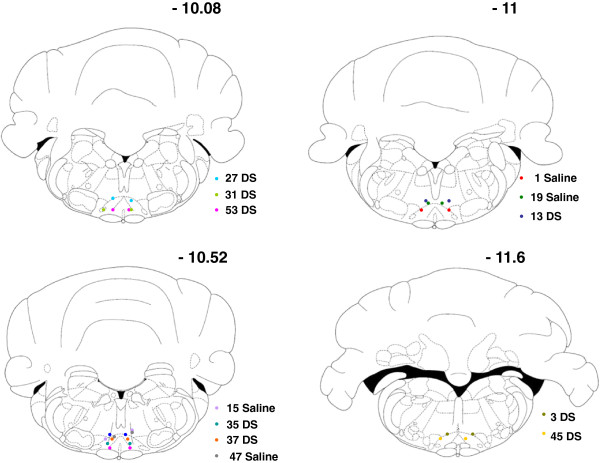
**Confirmation of bilateral microinjection sites of animals used in microarray experiment.** Only animals from the dermorphin-saporin + CFA group (dots labelled DS) and saline + CFA group (dots labelled saline) are shown. The numbers indicate the approximate anterior-posterior distance from Bregma in mm.

To determine the contribution of the descending MOR+ pathway to mechanical hypersensitivity following ankle joint inflammation, animals were divided into two groups, receiving intra-RVM injection of dermorphin-saporin (1.5pmole) or saline. The dose used was modified from the 3pmole described by others previously [21-23], and similar to a recent study that used a dose of 1.8pmole dermorphin-saporin in a study of stress induced hyperalgesia [27] as 3pmole doses of dermorphin-saporin were found to produce non-specific damage to brainstem neurons. Pilot experiments were carried out to assess the efficacy of dermorphin-saporin in specifically ablating MOR+ cells 28 days after injection into RVM The RVM region (n = 3 dermorphin-saporin treated group, and n = 3 from saline injected controls) were sectioned and stained for MOR. Counting of MOR+ cells was carried out manually using an immunofluorescence microscope. As expected, dermorphin-saporin microinjection led to a significant decresase in MOR+ cell number compared to saline injected controls. A decrease in MOR+ cell numbers in the 1.5pmole group (155 ± 4) was found when compared with saline control (208.7 ± 14.2) (independent samples t-test, p = 0.022, Figure 3B). Representative immunohistochemistry from dermorphin-saporin and sham treated animals are shown in Figure 3A. To confirm that the cell depletion was specific to MOR+ neurons, NeuN double labelling was carried out in a separate set of sections. High power single plane confocal images (Figure 3C), indicate the presence of surviving NeuN+, MOR- cells in dermorphin-saporin treated animals. This confirms specificity of the depletion for MOR expressing neurons.RVM lesioned rats were further subdivided into groups that underwent CFA injection or sham procedure. Paw withdrawal thresholds were measured prior to dermorphin-saporin or saline treatment, and monitored up to 7d post CFA injection. Three-way ANOVA with repeated measures was used to analyse the ipsilateral paw withdrawal thresholds. All CFA treated animals showed an increase in mechanical hypersensitivity on the ipsilateral hindpaw (p < 0.001, F(1,25) = 228.1). A subsequent two-way ANOVA with repeated measures was carried out on the two CFA treated groups, from 1d to 7d post CFA, and it was found that there was an overall main effect of dermorphin-saporin treatment on paw withdrawal thresholds from 1d to 7d post CFA injection (p = 0.015, F (1, 25) = 6.8). LSD post hoc analysis indicated significant differences in CFA groups at 1d, 3d, and 6d (Figure 4A).Paw withdrawal thresholds of the contralateral hindpaw were also measured. No main effect of CFA injection was identified, indicating contralateral mechanical sensitivity was unaffected by CFA injection (Figure 4B).At the end of the behavioural experiment, animals were culled and the dorsal quadrants of the lumbar spinal cord were dissected for microarray experiments. The brains of these animals were also removed, sectioned and stained using DAPI to identify the needle tracts. The approximate anterior-posterior, dorsal-ventral and lateral coordinates for the visible needle tracts were noted for all the animals from this experiment and the approximate locations in the CFA groups are shown (Figure 5). For all other animals it was found that at least one of the bilateral injections was within or close to the dorsal-ventral boundary of the RVM. Similarly the anterior-posterior coordinates were satisfactory for all animals (within −10.5 mm to −11.8 mm from Bregma).

### Characterisation of dorsal horn genes regulated by the MOR+ cell pathway at 7d post CFA

Gene expression profiles of the dorsal horn of rats lesioned with dermorphin-saporin were compared to saline injected control animals, at 7d following ankle injection of CFA. Following Limma testing, the non-adjusted p-value was used for ranking purposes, as described previously [28]. Only those genes with a non-adjusted p value of < 0.05 were considered as differentially regulated (2616 transcripts in total). Genes without an official gene symbol were also removed (leaving 1668 transcripts in total). The remaining transcripts were then ranked by fold change. An arbitary fold change cut-off of 1.2 was set, and this resulted in a final list of 129 genes. Additional file 1: Table S1 lists all of these genes with their ranking on the list based on fold change. The majority of the genes (76%) were downregulated in the dermorphin-saporin group.

**Figure 6:**
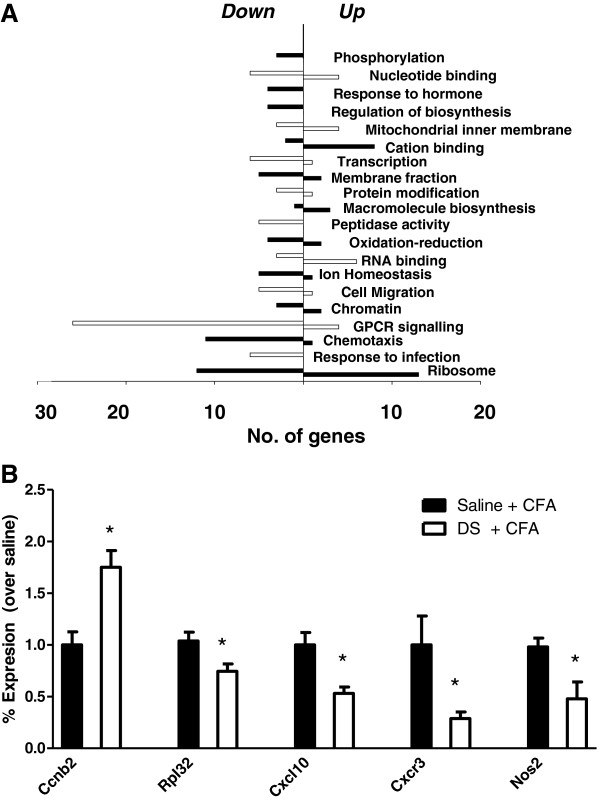
**DAVID bioinformatics and RT-qPCR validation. A).** 20 functional annotation clusters were identified using the DAVID online bioinformatics tool. The horizontal bars indicate the number of genes that are upregulated (to the right) or downregulated (to the left) in each cluster. Joined bars indicate the upregulated and downregulated genes for each cluster. For clarity, alternate black and white bars are used between clusters. For details of the genes contained in each cluster see Additional file 2: Table S2. **B).** A number of genes were selected for validation by RT-qPCR. 2∆Ct values were obtained for each group, using actin as the housekeeping gene. Data is presented as fold change of the dermorphin-saporin (DS) + CFA group over the saline + CFA control group, mean ± SEM. n = 4 – 6 per group, *p < 0.05 independent samples t-test.

To identify biological functions over represented within this list, DAVID bioinformatics software was used. This is a freely available online tool (http://david.abcc.ncifcrf.gov) and it was used to perform functional annotation of the genes in the list based on the gene ontology (GO) database. This consists of annotations of gene function using defined GO terms in three areas: biological process, cellular compartment and molecular function [29]. This analysis produced a list of 35 GO terms significantly enriched within the list. Functional annotation clustering was then carried out, to consolidate groups of similar annotations to minimise redundancy in function [30]. This analysis resulted in the identification of 20 clusters of annotations enriched within the gene list (Figure 6A). Full lists of genes within each cluster are shown in Additional file 2: Table S2.

The most significantly enriched cluster (with the highest EASE score, see methods) corresponded to genes with ribosomal function. The annotations of this category refer to ribosomal structural proteins, as well as genes involved in translation and those with a cytosolic location. This included several large ribosomal subunit components (e.g. *Rpl32* and *Rpl21*). Also within this group was *Ccnb2*, which encodes the cell-cycle mediator cyclin B2. This was one of the highest ranking fold changes identified with a 1.85 increase in the dermorphin-saporin group.

A second significant cluster contains genes with annotations for defense response, which includes a number of genes involved in immune system function. Among these was *Nos2*, which encodes the enzyme nitric oxide synthase 2 (inducible nitric oxide synthase), which is implicated in inflammatory responses [31]. The next cluster contains genes associated with chemotaxis, which are also involved in immune system function and inflammatory responses. This cluster included the chemokines *Cxcl9* and *Cxcl10*. All of these immune system and chemotaxis related genes were downregulated in the dermorphin-saporin group, suggesting that immune cell activation in the dorsal horn may be reduced in the dermorphin-saporin group. Interestingly, the GPCR signalling cluster contained the chemokine receptor *Cxcr3* which binds the chemotaxis ligands *Cxcl9* and *Cxcl10*.

### Validation of selected ribosomal and immune system related genes by RT-qPCR

A number of genes identified by the microarray analysis were selected for validation by RT-qPCR. These were selected from the four most significantly enriched functional annotation clusters: ribosomal function, defence response, chemotaxis and GPCR signalling. For each selected gene, 4 – 6 samples per group (dermorphin-saporin + CFA v saline + CFA) were used for validation. Gene expression was normalised to the housekeeping gene, β-actin.The most statistically significant cluster (with the highest enrichment score) contained genes with ribosomal functions which may be of interest as translation is important in many forms of neuronal plasticity, including pain states. As an example of a gene from this cluster, downregulation of the ribosomal protein Rpl32, which had a fold decrease of 1.68 on the microarray, was confirmed by RT-qPCR (p = 0.0399, Figure 6B). Ccnb2, encoding the protein Cyclin B2, was one of the highest fold gene changes identified by the array, with a 1.85 fold increase in the dermorphin-saporin treated animals and ranking number 4 on the list. It was also among the minority of genes that were upregulated in the dermorphin-saporin group. This increase was confirmed by RT-qPCR, with a significant increase in expression found in the dermorphin-saporin + CFA treated group compared with saline + CFA controls (p = 0.0041, Figure 6B).

The next most significant clusters, defence response and cheomotaxis, contained a number of immune system related genes. *Nos2*, encoding the enzyme nitric oxide synthase 2 (inducible nitric oxide synthase, iNOS) has been shown to play a role in central sensitisation in both inflammatory and neuropathic pain states [32-35]. *Nos2* was found to be downregulated in the dermorphin-saporin group. RT-qPCR confirmed a significant decrease in *Nos2* expression in the dermorphin-saporin group (p = 0.022, Figure 6B).

*Cxcl10* was among the genes in the chemotaxis cluster. This is a member of the CXC family of chemokines which are induced by IFN-y. CXCL10 is a ligand for the G-protein coupled chemokine receptor *Cxcr3*[36] which was also identified on the microarray. *Cxcl10* and *Cxcr3* were downregulated in the dermorphin-saporin group in the microarray analysis, and RT-qPCR confirmed a significant decrease in all both genes (*Cxcl10* p = 0.0132, *Cxcr3* p = 0.0379, Figure 6B) Although these genes have been reported to be upregulated in the DRG in certain inflammatory conditions [37,38], the present study is the first to identify regulation this chemokine family within the dorsal horn.

## Discussion

Few studies to date have addressed the role of the descending system in joint pain, except for a number of electrophysiological studies of descending inhibition, and only in the acute phase of the pain state [39-41]. Here we have combined lesion of the RVM with behavioural studies to address the role of descending facilitation in a model of inflammatory joint pain. In addition microarray analysis has led to the identification of a number of possible mediators of descending facilitation at the level of the dorsal horn.

We first investigated the role of the descending 5-HT pathway in the development of mechanical hypersensitivity following ankle injection of CFA. Previous studies have indicated the descending 5-HT pathway contributes to the maintenance of behavioural hypersensitivity in neuropathic pain [26]. In the present study intrathecal administration of the selective toxin 5,7-DHT attenuated mechanical hypersensitivity at 1d and 2d post CFA injection, suggesting that the 5-HT pathway contributes in a time-dependent manner to hypersensitivity in this pain state. In the hours following inflammation and at later time points, the 5-HT system does not appear to facilitate the pain state. This supports previous findings by others that siRNA silencing of the tryptophan hydroxylase (TPH) enzyme within the RVM attenuates mechanical hypersensitivity at 1d – 3d following plantar injection of CFA [24].

Our second lesion experiment targeted the electrophysiologically defined ON cells of the RVM, which are believed to be facilitatory in nature [42]. Although ON cells do not have a single neurochemical classification, they can be identified by their direct responsiveness to morphine implying that they express the mu opioid receptor (MOR) [19,20]. This characteristic has been exploited previously by others to ablate the MOR expressing (MOR+) neurons of the RVM using the selective neurotoxin dermorphin-saporin [21-23]. We found that, as with 5,7-DHT treatment, MOR+ cell depletion attenuated mechanical hypersensitivity. However this effect was more prolonged than for 5,7-DHT depletion, with significant attenuation observed from 1d to 7d post CFA injection. This increased attenuation may have been because some MOR positive neurons are also 5-HT positive [20] and lesions of RVM therefore would have caused partial ablation of both descending control pathways.

However while both the 5-HT and MOR+ cell pathways played a role in regulating the mechanical hypersensitivity associated with joint inflammation, the magnitude of attenuation in both lesion studies was considerably smaller than that observed in neuropathic pain states [21-23]. Similarly the time of onset of descending facilitation differs in the present study than that shown previously for neuropathic pain. The previous dermorphin-saporin studies of neuropathic pain indicated that descending facilitation is required for the maintenance but not the induction of behavioural hypersensitivity [21-23]. In contrast, descending facilitation of joint pain is evident from one day onwards. This suggests that in neuropathic injury, descending facilitation is required for the maintenance of the pain state, whereas in joint inflammation descending facilitation contributes to both the induction and maintenance phases. It should be noted that we have measured mechanical hypersensitivity of the hindpaw, which is a reflection of secondary hyperalgesia. We did not address primary hyperalgesia of the inflamed joint, which would require measurement of load bearing and other more direct measures of primary sensitization. Nonetheless, the present findings support previous studies which suggested that descending facilitation modulates secondary hyperalgesia [43-45].

While the contribution of descending facilitation to central sensitisation and behavioural hypersensitivity in a variety of pain states has been extensively studied [21,22,24,25] little is known about the molecular mechanisms underlying descending facilitation. Microarray analysis has however been used to characterise changes in dorsal horn gene expression at 2 h-7d following ankle injection of CFA [28]. To build on these findings, the present study aimed to examine the contribution of descending facilitation to the regulation of gene expression at the 7d time point. Microarray analysis was carried out on two groups, both with CFA inflammation, and with dermorphin-saporin or saline pretreatment as the only variable. In this way the analysis revealed genes that are regulated in this pain state by the MOR+ RVM descending pathway.

A number of cellular mechanisms could potentially contribute to descending facilitation of dorsal horn excitability. Changes in receptor availability, presynaptic release of neurotransmitters, post-synaptic changes in intracellular signalling pathways, transcriptional regulation and immune cell activation are all key features of central sensitisation [46] and could be subject to modulation by descending facilitation. However, surprisingly few genes with neuronal annotations were identified in our analysis, while many genes associated with immune cell function were identified.

A prominent role for immune cells in dorsal horn in mediating central sensitisation and behavioural hypersensitivity has been established [16,17,47,48] and immune system related genes are a feature of many other microarray studies of the dorsal horn [47-49]. Many of the immune related genes identified by our microarray analysis were found to be downregulated by dermorphin-saporin treatment, suggesting that descending facilitation by the MOR+ cell pathway is a positive modulator of immune cell processes within the dorsal horn.

Among the genes confirmed by RT-qPCR was *Nos2,* which encodes the enzyme inducible nitric oxide synthase (iNOS). *Nos2* gene expression is induced in peripheral immune cells during inflammation, and has also been shown to be regulated in microglia and astrocytes [50]. Nitric oxide (NO) itself has been implicated in central sensitisation in various pain states [32,51-55]. Although the focus has largely been on neuronal NOS as the enzyme responsible for spinal production of NO, a number of reports have implicated iNOS protein in inflammatory and neuropathic pain states [32-35]. Bilateral induction of *Nos2* in the dorsal horn has been shown to occur following ankle injection of CFA [33] and our present data suggests that *Nos2* expression may be modulated, at least in part, by descending pathways. Interestingly upregulation of *Nos2* occurs in the spinal cord in a model of stress-induced hyperalgesia, and CCK signalling within the RVM is required for this process [56]. Our finding also suggests that *Nos2* gene expression in the dorsal horn is modulated by descending pathways.

One of the key findings of our present study was the downregulation of two members of the same family of chemokines in the dermorphin-saporin treated group. The expression of these genes was not altered by CFA joint inflammation alone [28]. Chemokines are small molecular weight proteins which regulate leukocyte migration and activation and act as a key component of the immune response [57]. Within the dorsal horn, a number of chemokines have been shown to play a role in various pain models and there is an established role for chemokines such as CX3CL1 (fractalkine) and CCL2 within the dorsal horn in both neuropathic and inflammatory pain states [16]. *Cxcl9* and *Cxcl10* are members of the interferon gamma (IFN-γ) inducible subset of CXC chemokines. A number of previous studies have suggested a role for the cytokine IFN-y within the dorsal horn in chronic pain states [58-61]. As targets of IFN-y signalling, *Cxcl9* and *Cxcl10* may therefore be involved in nociception in the dorsal horn. Notably the shared receptor for these chemokines, *Cxcr3,* was also downregulated in the dermorphin-saporin treated group. The simultaneous downregulation of these *Cxcl9*, *Cxcl10* and *Cxcr3* suggests that this chemokine-receptor signalling pathway may contribute to descending facilitation via the MOR+ cell pathway, indicating a novel role for this chemokine family in pain signalling within the dorsal horn.

Although most studies of CXCL10 in the CNS have focused on its expression in immune cells and role in neuroinflammation [36] one study has demonstrated that CXCL10 is expressed constitutively in neurons in culture, and may be released tonically at low levels [62]. Furthermore exposure of hippocampal neurons *in vitro* to exogenous CXCL10 causes increased excitability [63]. Although to date, chemokines within the CNS have been investigated predominantly as modulators of immune cells, in future it may be of interest to investigate their roles in neuronal transmission [64].

The expression of CXCL9 and CXCL10 is also increased in the joints of patients with rheumatoid arthritis [65,66] suggesting that these chemokines may contribute to the joint pathology associated with the disease. Indeed CXCR3 receptor antagonists have also been shown to be effective in decreasing the pathogenesis of rheumatoid arthritis in an animal model [67]. A pronociceptive role for CXCL9, CXCL10 and CXCR3 signalling within the dorsal horn would provide further support for the use of chemokine antagonists in the systemic treatment of joint pain states.

In conclusion, the present data demonstrates for the first that time that descending facilitation contributes to behavioural hypersensitivity following joint inflammation. This implies that in addition to targeting the underlying joint pathology, patients suffering from joint pain symptoms may benefit from treatments that reduce central sensitisation and descending facilitation. In particular, the CXC family of chemokines are subject to regulation in the dorsal horn by descending facilitation and therefore may be promising targets for further studies.

## Methods

### Animals and inflammatory joint pain model

All studies used male Sprague–Dawley rats supplied by the Biological Services Unit at University College London. All procedures complied with the United Kingdom Animals (Scientific Procedures) Act 1986. Rats were housed in cages in groups of 1 – 4 animals with a 12 h light–dark cycle (lights on at 8.00 am). Access to food and water was *ad libitum*. Joint inflammation was carried out by injection of 10 μl Complete Freund’s adjuvant (CFA, Sigma) as described previously [28]. Sham animals were exposed to the same anaesthesia as the CFA treated group but received no injection.

### Intrathecal administration of 5,7-DHT

Intrathecal injection of 5,7-dihydroxytryptamine was carried out as described previously [25]. Animals (weighing 180 g – 200 g at the time of injection) were pre-treated with 25 mg/kg i.p. desipramine hydrochloride (Sigma) dissolved in saline to protect against noradrenergic toxicity one hour prior to surgery. Animals received 10 μl of 5,7-dihydroxytryptamine (5,7-DHT) dissolved in saline (6 μg/μl, Fluka), or saline only. Animals were allowed to recover for 6d prior to ankle injection of CFA. The numbers per group for this behavioural experiment were 5,7-DHT CFA (n = 6), saline CFA (n = 6), 5,7-DHT sham (n = 7), saline sham (n = 7).

### Microinjection of dermorphin-saporin to the RVM

Animals (weighing 200 – 250 g at the time of microinjection) were secured using a stereotaxic frame (Kopf instruments), under isoflurane anaesthesia as described above. A dental drill was used to form a single hole at the site of microinjections. Two microinjections (volume 0.5 μl each) were carried out using a Hamilton syringe at the following coordinates: anterior-posterior: −10.5 mm from Bregma, lateral: ± 0.6 mm, and dorso-ventral -9 mm. Dermorphin-saporin (Advanced Targeting Systems, 1.5pmole/μl, prepared in saline) was dispensed over the course of 5 minutes. The needle was held in place for 1 min following injection. The skin was sutured using 5–0 Mersilk. Animals were allowed to recover for 28 – 35d prior to ankle injection of CFA. The numbers per group for the behavioural experiment were dermorphin-saporin CFA (n = 8), saline CFA (n = 8), dermorphin-saporin sham (n = 7), saline sham (n = 6).

### Behavioural testing

On the day of testing, animals were transported to the testing room in their home cages, and placed in clear plastic containers on a wire mesh floor. Animals were left to settle for 15 min prior to testing. Habituation to the testing environment was achieved by taking a series of baseline measures, once daily for at least 3d prior to the initial experimental manipulation. The mechanical paw withdrawal threshold was obtained for the ipsilateral and contralateral hindpaw of the animal. A series of calibrated von Frey filaments (Stoelting) were applied to the centre of plantar surface of the hindpaw, between the footpads. The hairs were applied in ascending order, with 0.07 g as the minimum and 60 g as the maximum cut off point. Each von Frey filament was applied until bending occurred. The filament was held in place for approximately 4 s. For each filament the stimulus was applied five times at 5 s intervals. The paw withdrawal threshold was defined as the lowest weight von Frey filament at which a brisk withdrawal was observed at least once out of the five repeated stimuli. When a withdrawal response was observed to a given filament, no further filaments were applied. The ipsilateral paw withdrawal thresholds for each animal were obtained first, followed by repetition of the procedure on the contralateral paw. The experimenter was blind to treatment group of the animal in all cases.

### Immunohistochemistry

For immunohistochemistry experiments, rats were deeply anaesthetised with pentobarbital (Euthatal, 0.5 – 1 ml per animal, i.p.) and perfused transcardially with saline containing 5000 IU/ml heparin, at a rate of 30 ml/min for 4 min. This was followed by 4% paraformaldehyde (PFA) in 0.1 M phosphate buffer (PB) for 8 min (approximately 240 ml). Brains and spinal cords were dissected and post fixed in 4% PFA for 2 h. Tissue was then cryoprotected in 30% sucrose (in 0.1 M PB) and stored at 4°C until sectioning. 40 μm sections of the lumbar spinal cord (L4 – L6) or RVM (from approximately −10.3 to −11.3 mm from Bregma) were obtained using a freezing microtome (Leica) and stored in 5% sucrose (in 0.1 M PB) at 4°C until staining.

Immunohistochemistry was carried out on freely floating sections. Sections were blocked for one hour in 1 ml 0.1 M PB, 30 μl goat serum (Vector) and 30ul 10% Triton X (Sigma) followed by incubation with primary antibodies in tris-triton buffered saline (TTBS) overnight at room temperature (5-HT, 1:75, Chemicon (MAB342); mu opioid receptor, 1:10,000, Neuromics (RA10104)). This was followed by the appropriate biotinylated secondary antibodies at a concentration of 1:400 for 90 min at room temperature. Sections were then incubated with avidin biotin complex (ABC Elite; 1:250 Vectastain A plus 1:250 Vectastain B; Vector Laboratories, Burlingame, CA) for 30 min. For MOR staining, this was followed by a signal amplification step with biotinylated tyramide solution (1:75 for 7 min; PerkinElmer, Wellesley, MA). 5-HT immunohistochemistry did not include the tyramide signal amplification step. Finally, sections were incubated with FITC-avidin for 2 h (1:600, Vector). For MOR double labelling, the sections were incubated overnight at room termperature with the NeuN primary antibody (1:1000, Cell Signalling). The sections were incubated with the direct Alexa Fluor 594 anti-mouse antibody (1:500, Invitrogen) for 2 h. Sections were mounted on gelatin coated slides in 0.1 M PB, followed by drying at room temperature and coverslipping with fluoromount (Sigma).

Fluorescence labelled sections were viewed using a Leica DMR microscope (Leica Microsystems). Images were acquired using a Hamamatsu CCD digital camera (C5985, Hammamatsu Photonics) using Openlab 4.0.4 software (Improvision). MOR+ neurons in the RVM were counted manually. The RVM was defined by the boundaries of the NRM and GiA, from approximately −10.3 mm to −11.3 mm from Bregma, according to the rat brain atlas [68]. Counts were carried out while blind to treatment group. All sections per animal were counted, and the the 5 sections with the highest number of MOR+ neurons, per animal, were used for analysis. These were summed to generate a total number of positive cells per animal (n = 3 per group), and data was plotted as the mean ± SEM per group.

For MOR/NeuN double labelling, a laser scanning confocal microscope (Leica TCS SPE) was used. Sequential laser channel acquisition was used and all images were analysed as single z-planes from each channel, and with a merged image of both channels.

### Tissue collection and RNA extraction

Rats were were killed by CO₂ asphyxiation. The spinal cord segment corresponding to the lumbar L4-L6 region was rapidly dissected on ice, and the ipsilateral and contralateral quadrants of the superficial dorsal horn separated using a blade. The dorsal horn quadrants were homogenised by hand in 700ul Qiazol (Qiagen) for 1 to 2 min. The sample was removed and passed through a Qiashredder column and spun in the centrifuge at 14,000 rpm for 2 min. The sample was then left at room temperature for 5 min before the RNA was extracted using the Qiagen RNeasy kit. 140 μl chloroform was added to the sample and the tube was capped and shaken vigorously for 15 s. The samples were then left at room temperature for 3 min followed by centrifugation at 12,000 rpm at 4°C for 15 min.

The upper aqueous phase was transferred to a new tube, with care taken to minimise cellular debris remaining in the sample. 53% ethanol was added to the removed aqueous phase. This was mixed well by pipetting up and down, and added to the RNeasy spin column, and centrifuged to remove the ethanol. A wash step using buffer RW1 (from the RNeasy kit) was carried out before adding DNAase I incubation mix (Qiagen) to the column for 15 min. A series of further washes using buffer RW1 and RPE from the RNeasy kit were then performed, before a final drying step and elution of the RNA in 25ul of RNase free H₂O. 1 μl of each RNA sample was applied to a Nanodrop spectrophotometer and the concentration of RNA per sample was determined in ng/μl. The contamination levels of organic solvents (indicated by a low 260/230 value) and protein contamination (indicated by a low 260/280 value) were also determined using the Nanodrop. RNA was then stored at −80°C until further use.

### Microarray study

For the microarray RNA was extracted from the ipsilateral dorsal horn from two groups of animals. One group had dermorphin-saporin lesion of the RVM and the other saline control injections. 28 days later both groups received a unilateral intraarticular injection of CFA (as above). Tissue was taken for for microarray analysis 7 days after CFA injection, n =5 in both groups. RNA samples were normalised to a concentration of 50 ng/μl in RNase free H₂O before submission to the UCL genomics service. Complementary DNA (cDNA) synthesis was carried out using the Ambion WT expression kit. This was followed by labelling of the cDNA using the Affymetrix WT terminal labelling kit. Labelled cDNA was then hybridised to Affymetrix rat gene 1.0 ST arrays and the intensity of fluorescence of the arrays was measured using an Affymetrix gene chip scanner.

The raw data was obtained in the form of .CEL files, which contain the results of the intensity calculations per chip. Analysis of the raw data was carried out in R, a language and environment for statistical computing and graphics (http://www.r-project.org/), and the Bioconductor plugin for microarray analysis was used (http://www.bioconductor.org/). Limma testing was used to assess differential expression between the two treatment groups. The adjusted p values were not significant for any of the probe sets, and for further analysis, the non-adjusted p value was used. Previous published work by our group has also used the non-adjusted p value [28].

The online bioinformatics tool DAVID (http://david.abcc.ncifcrf.gov) was used to identify clusters of genes within the list with statistically overrepresented functional annotations [30].

### RT-qPCR

Complementary DNA (cDNA) was synthesised from the RNA samples. The reaction was carried by adding 0.5 μg RNA sample to a mix of 0.2 μl Oligo dT20 (Invitrogen), 0.8 μl random nonamers (Sigma), 1 μl PCR nucleotide mix (Promega) and bringing the reaction volume to 13 μl by adding RNase free water. This was then incubated at 65°C for 5 min. The samples were then quickly chilled on ice for 5 min. The tubes were briefly centrifuged to collect the mixture, and 4 μl first strand buffer (Invitrogen), 1 μl DTT (Invitrogen), 1 μl RNaseIN ribonuclease inhibitor (Invitrogen) and 1 μl of the key reaction component, reverse transcriptase (Superscript III, Invitrogen) were added. This was gently mixed by pipetting up and down and the samples were then incubated at 25°C for 5 min, 50°C for 50 min and 70°C for 15 min. The cDNA samples were then placed quickly on ice before storage at −20°C until use in RT-qPCR assays.

Oligonucleotide primers were designed to target selected genes based on the Affymetrix probe sequences (Sigma). A list of primer sequences is given in Table 1. RT-qPCR assays were carried out on 96 well plates, with cDNA samples run in triplicate and controls for master mix contamination (no cDNA added) and cDNA contamination (negative control from the cDNA synthesis, without transcriptase added) included in each plate. A master mix solution was prepared for each plate so that each well contained 12.5 μl SYBR green (Sigma), 9.5 μl RNase free H₂O, and 1 μl stock solution of the forward and reverse primers of the target gene. 1 μl cDNA was added to the wells after they were filled with the master solution.

**Table 1:** Sequences of primers for RT-qPCR

**Gene name**	**Primer**	**Sequence**
Cyclin B2	Ccnb2F	CTAAGAGCCATGTGACTGTC
	Ccnb2R	CAGAACTGTAGGTTTCGG
Chemokine (C-X-C motif) ligand 10	Cxcl10F	ATACTCACAGGAACCTAGACAT
	Cxcl10R	CCATCCAACACATCTTGTAATATG
Ribosomal protein L32	Rpl32F	GTTCATCAGGCACCAGTC
	Rpl32R	TGACATCGTGGACCAGAA
Chemokine (C-X-C motif) ligand 9	Cxcl9F	GATGAAGCCCTTTCATACTGC
	Cxcl9R	GTGGTTGTGAGTTTTGCTCCAATC
Chemokine (C-X-C motif) receptor 3	Cxcr3F	AGCCCTCACCTGCATAGTTG
	Cxcr3R	GCCACTAGCTGCAGTACACG
Nitric oxide synthase 2	Nos2F	GATATCTTCGGTGCGGTCTT
	Nos2R	GGCCAGATGCTGTAACTCTT

The Sigma 3-step amplification protocol was used with a Bio-Rad CFX96 PCR C100 thermal cycler. Data was analysed using Bio-Rad CFX96 software. Ct values (threshold cycle, which is the number of cycles taken for the fluorescence labelling to increase above background) were obtained for each target gene. This value is inversely proportional to the log of the copy number of the cDNA in the sample. Actin was used as a housekeeping gene. Relative gene expression was calculated using the 2ΔCt method, where the data for each gene is expressed in the form 2 to the power of (Ct Actin – Ct target). N = 4–5 in both groups.

### Statistical analysis

All data was analysed using SPSS (PASW) 18 (IBM). For behavioural experiments, logarithmic transformation (log2) was carried out on the paw withdrawal thresholds (g) before statistical analysis as Levene’s test for equality of variance was significant [69,70]. Analysis of variance (ANOVA) with repeated measures was used for all time course experiments. To proceed to further post hoc testing at least one main effect (CFA treatment or toxin) or interaction was required to be statistically significant (p < 0.05). Subsequent two-way ANOVA with repeated measures was carried out to determine if there was an overall effect of CFA or toxin across a specific time window. For cell count experiments and RT-qPCR experiments independent samples t-tests were used to compare means between two groups. In all tests a p-value of < 0.05 was deemed significant.

## Competing interests

The authors declare that they have no competing interests.

## Authors’ contributions

SPH designed and planned the study. FBC and SMG carried out the experiments and analysed the data. All authors contributed to the writing of the manuscript and approved the final version.

## Supplementary Material

Additional file 1: Table S1 List of regulated genes identified by microarray after statistical analysis. Click here for file

Additional file 2: Table S2 Results of functional annotation clustering. Click here for file
